# Dental Students' Knowledge of Oral Health for Persons with Special Needs: A Pilot Study

**DOI:** 10.1155/2015/568464

**Published:** 2015-04-09

**Authors:** Fouad Salama, Bader Al-Balkhi, Faika Abdelmegid

**Affiliations:** ^1^Department of Pediatric Dentistry and Orthodontics, College of Dentistry, King Saud University, Riyadh, Saudi Arabia; ^2^College of Dentistry, King Saud University, Riyadh, Saudi Arabia; ^3^Department of Oral Medicine and Diagnostic Sciences, College of Dentistry, King Saud University, Riyadh, Saudi Arabia

## Abstract

*Objectives*. The purpose of this pilot study was to assess the knowledge and awareness of dental students with respect to oral health care of the person with special health care needs (SHCN) and evaluate effectiveness of an education program on improving their knowledge. *Method*. An evaluation consisting of a questionnaire was answered before and immediately after a 30-minute educational presentation in the form of a DVD that includes a PowerPoint and a video of oral health care for individuals with SHCN. The questionnaire was based on the materials and information presented in the DVD and included 26 questions (true/false/I do not know). *Results*. The mean (±SD) score on the pretest was 10.85 (±5.20), which increased to 16.85 (±5.47) on the posttest. This difference was statistically significant (*P* < 0.001). Forty percent of the students surveyed reported that they were very satisfied with the educational part of the presentation, while 50% were somewhat satisfied. Thirty percent of students expressed that the educational intervention used is very effective. *Conclusions*. Viewing the educational intervention was effective in informing the sophomore students and providing them with instructive basic information on person with SHCN. Dental colleges should increase students' knowledge, training, and exposure to individuals with SHCN.

## 1. Introduction

Several definitions for special needs and disabilities exist worldwide with no agreement on a standard definition being recognized [1–4]. The World Health Organization [3] defines disabilities as an umbrella term which includes activity limitations, impairments, and participation restrictions. An activity limitation is a struggle faced by person in performing a job or action while impairment is difficulty in function or structure of the body [3]. Also, children with special health care needs were defined as* “those who have or are at increased risk for a chronic physical, developmental, behavioral, or emotional condition and who also require health related service of a type or amount beyond that required by children generally”* [4]. In 2004, the American Academy of Pediatric Dentistry [5] embraced a very similar definition. This definition was later revised in 2012 as* “any physical, developmental, mental, sensory, behavioral, cognitive, or emotional impairment or limiting condition that requires medical management, health care intervention, and/or use of specialized services or programs”* [1].

Many countries are using International Classification of Functioning, Disability and Health (ICF) in their national disability surveys [6]. Estimated prevalence rates on disability vary widely across countries [7]. The World Health Survey conducted in 2004 by WHO on 59 countries reported that the average prevalence of disability in adult population was 15.6% (4.2 billion people), ranging from 11.8% in higher income countries to 18% in lower income countries of whom 2–4% experience substantial difficulties in functioning (WHO 2011 [3]). In Saudi Arabia, a field survey among children in Saudi Arabia in 1997–2000 was carried out and 6.33% of the sample were recorded as disabled [8]. The region with the highest proportion of disability children was Jazan (9.90%) and Riyadh had the lowest (4.36%). Motor disability was the most common kind of disability (3.0% of the total sample), followed by learning disability (1.8%) [8]. The highest proportion of disability was found among children with disabled parents [8]. Other authors reported that in children 0–18 years in Saudi Arabia, the occurrence of mental retardation was 8.9 per 1000 children and moderate or severe retardation was classified in 70.9% of these children and 83.2% were not joining school [9]. Nearly 1 in 5 families in the United States have a child with SHCN and many of them for one reason or another are not able to perform independent oral health care and depend on another family member or caregiver to provide the necessary oral hygiene care [10]. In United States, about 12.5 million or approximately 16 to 18 percent of children have a SHCN [4, 10, 11]. A more recent report indicated that nearly 10.2 million children are SHCN under the age of 18 years [12].

Children with SHCN are amongst the most underserved in the population, having more dental diseases than any other segment of the population, and are more likely than other children to have not fulfilled their oral care needs [12–15]. Other national surveys and recommendations have shown that up to 8% of persons with SHCN do not receive routine preventive dental care [16–18]. Persons with SHCN must overcome many barriers to procure the health care they need and a recent report shows that even with private insurance and well-educated parents, nearly 20% of the studied children with SHCN had unmet dental care needs [19]. While the access to care issue for person with SHCN is of great concern and difficulty for their parents, it should also urge all health care workers to the unmet dental needs which may lead to serious infections which can further complicate their overall health status [14]. With increasing number of children with SHCN and their unmet dental care needs, the dental community is making little progress to increase the access to much needed oral health services for them [19].

Education of dental students provide an opportunity to help improve and increase oral care of person with SHCN, and dental schools would get advantage by exploring different models to instruct and teach dental students on the best way to deal with and address oral care and health of person with SHCN [17, 20, 21]. Children and adults with SHCN are amongst the most underserved and have more dental diseases than any other segment of the population. Therefore, the need for an educational component of a preventive oral health care program for person with SHCN is important. However, research on educational approaches related to person with SHCN is limited. In this pilot study, we hypothesized that viewing a precise, educational presentation with video would provide dental students with educational and useful information as well as a directed approach to person with SHCN. Therefore, the purpose of this study was to assess the knowledge and awareness of a group of sophomore dental students with respect to oral health care of person with SHCN and evaluate the effectiveness of an education program on improving their knowledge.

## 2. Materials and Methods

This study, including the pretest questionnaire, the DVD educational intervention, and the posttest questionnaire, was approved by College of Dentistry Research Center (CDRC) and Ethics Committees, College of Dentistry, King Saud University. This study involved educating sophomore dental students about oral health care of person with SHCN and assessing their knowledge before and after educational intervention in a nonequivalent control group design. The educational intervention and questions were generated based on reviews of the pediatric and general dental literature as well as the AAPD guideline on management of dental patients with special health care needs to provide an informative and concise representation designed to educate sophomore dental students regarding person with SHCN.

An evaluation consisting of a questionnaire was answered before and immediately after a 30-minute educational presentation in the form of a DVD that includes a PowerPoint and a video of oral health care for individuals with SHCN (interested readers may contact the corresponding author).

Pretest questionnaire was completed by each student and was collected by the investigators and placed in an envelope. Participants were then asked to watch the educational intervention. This intervention was in the form of a DVD which included a PowerPoint presentation and a video clip with information on important aspects of delivering oral care for person with SHCN. After the educational intervention the study participants were given the posttest questionnaire. The questionnaire was based on the materials, information, and content viewed in the DVD and included 26 questions (true/false/I do not know) which were given before and immediately after the educational approach and intervention. The pretest questionnaire included demographic information and questions regarding if they have had any training informal or formal in oral health care for person with SHCN before the presentation and how would they rate their knowledge of oral health care for person with SHCN. Six questions were added to the posttest which included questions on the digital presentation's helpfulness in refining their understanding of oral care for person with SHCN, participant satisfaction, digital presentation effectiveness, likely hood of using the information in the presentation, and extent of knowledge of oral care for person with SHCN after the presentation. Validation of the survey and questions were tested before in a different unpublished study. The survey and questions were reviewed and tested by three pediatric dentists and modifications to the survey and questions were made based on their review. Also, the survey was pilot tested for test-retest reliability and clarity of the questionnaire by randomly selecting 10 of the target participants who were not included in the main study. Accordingly revision of the questionnaire was performed to avoid misinterpretation of the questions. Students were asked not to put their name or any other information on the questionnaires that could identify it as theirs. The pre- and posttest were identified by the same distinctive number for the same student, that is, anonymous. The students were asked to stay in the same seat during the administration of the research survey to facilitate distribution and collection of the pre- and posttest questionnaires.

Statistical analysis in form of frequency and percentage of individual score and total scores of the knowledge test were calculated and the difference in correct answers of pre- and posttest scores was compared using paired *t*-test. All statistical analyses were set with a significance level of *P* < 0.05. The statistical analysis was carried out with SPSS V16.0 (Statistical Package for the Social Sciences, SPSS, Chicago, Ill).

## 3. Results

The average (±SD) score on the pretest was 10.85 (±5.20), which increased to 16.85 (±5.47) on the posttest. This difference was statistically significant (*P* < 0.001). Frequency of the answers in the pre- and posttest questionnaires is presented in Table 1. The most difficult question (highest wrong answer) in pretest was related to useful use of a log book of what works and what does not work when designing an oral hygiene regimen for the person with SHCN by the caregivers, where the wrong answer was 14 (70%). The same question was the most difficult question in posttest where the wrong answer was 16 (80%) which indicated no improvement. The easiest question (highest correct answer) in pretest was question number 17, asking if dry mouth can contribute to tooth decay and gum disease, where the correct answer was 17 (85%), while the easiest questions in posttest were questions numbers 1 and 4, asking if persons with SHCN have a higher prevalence of conditions that contribute to poor oral health and if pica is a behavior sometimes observed in a person with SHCN, where the correct answer was the same for both questions, 19 (95%). In the pretest, the total number of the answers where the students reported that “they do not know” was 190. This number was reduced to 59 in the posttest results. The highest answers for the questions as “I do not know” in pretest was in questions numbers 4, 11, and 15, asking if pica is a behavior sometimes observed in a person with SHCN, if a person who has spina bifida is especially prone to developing an allergic reaction or sensitivity to local anesthetic, and if GERD is a condition in which patients vomit in order to rechew it, where the answer was the same for those questions, 14 (70%). The answers to those same questions were 0 (0%), 5 (25%), and 4 (20%) in the posttest indicating substantial improvement.

Two (10%) students reported having formal training on oral health for persons with SHCN which was in the form of dental behavior and oral medicine courses, while three (15%) students reported having informal training on oral health for person with SHCN which was in the form of Internet information and some YouTube videos.

All dental students participating in the study were requested to rate their knowledge of oral health for person with SHCN on the pre- and posttest questionnaire. The answers to the question “how would you rate your knowledge of oral health for person with SHCN” before and after the presentation/educational intervention are presented in Table 2. Only one student (5%) rated his knowledge as moderate, 12 (60%) rated their knowledge as minimal, and 7 (35%) said they had no knowledge of how to provide oral care for person with SHCN on the pretest questionnaire. These ratings improved after intervention and became 1 (5%) extensive, 16 (80%) moderate, and 3 (15%) minimal, and no one said they had no knowledge of how to provide oral care for person with SHCN on the posttest questionnaire.

Eight (40%) of the dental students surveyed reported that they were very satisfied with the educational component of this presentation, 10 (50%) expressed that they were somewhat satisfied, and 2 (10%) reported that they were not satisfied. Six (30%) of the dental students described the educational intervention used in this study to be very effective, while 13 (65%) reported that the educational intervention was somewhat effective. Nineteen (95%) dental students who contributed and joined the study reported on the posttest questionnaire that the educational intervention was helpful in improving and refining their understanding of oral health care for person with SHCN. Of the dental students who completed the posttest questionnaire, 9 (45%) reported that they were very likely to practice and use the information provided in the educational intervention while 9 (45%) responded that they were somewhat likely to practice and use the information provided in the educational intervention. Two (10%) reported that they were not likely to practice and use the information provided in the educational intervention.

**Figure 1:**
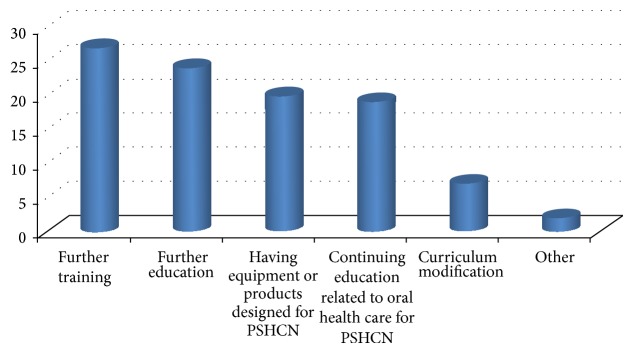
The answer to the question: which of the following would improve your ability to provide oral health care for persons with special needs?

When asked what they would like to change about the presentation, 12 (60%) said there is nothing they would change in the presentation while 8 (40%) have suggested changes on the presentation. Among the 40% who suggested changes on the presentation, 1 (5%) said PowerPoint slides should be explained as he does not like reading, 6 (30%) said it is fast, 4 (20%) wished the presentation provided more information and videos, and 2 (10%) wanted to add ways to deal with person with SHCN in the dental clinic. For the question “which of the following would improve your ability to deliver oral health care for persons with special needs?” student reported several selections (Figure 1) and, under others, 2 (10%) students would like to have clinical case presentations.

## 4. Discussion

Oral and general health care for a person with SHCN requires “*specialized knowledge acquired by additional training, as well as increased awareness and attention, adaptation, and accommodative measures beyond what are considered routine*” [1]. Pediatric dentists have taken a lead role in providing dental care to children with SHCN [5]. As such pediatric dental programs provide instruction and hands on training in providing comprehensive oral health care as part of a dental home for CSHCN [22]. Unfortunately with only approximately 5,000 practicing pediatric dentists in the United States it is clear that other dental professionals including dental students will be needed to care for children with SHCN [5]. It has been reported that few dental students experience treating persons with SHCN in dental school [23, 24]. In 2004 the Commission on Dental Accreditation adopted a new standard that dental students obligated to be competent in evaluating the treatment needs of person with SHCN [25]. The educational intervention in the present study increases the mean score on the pretest from 10.85 to 16.85 on the posttest and this difference was statistically significant. Also the total number of the answers where the students reported that “they do not know” in the pretest was 190 and this number was reduced to 59 in the posttest results.

In Saudi Arabia, a prevalence study for children with disability less than 15 years reported 42.8 per 1000 total with minor impairment and 3.76 per 1000 total population with major impairment [26]. So, the importance of person who provides oral health care for persons with SHCN including health care professionals, caregivers, students, and parents is critical. Also to make an effort to advance their knowledge and adequately support them with tools to help in taking better care of persons with SHCN. So, a well-prepared dental workforce including dental students is critical to improve the oral health of persons with SHCN [27]. Providing caregivers with the techniques and tools of preventive measures such as brushing the teeth of their persons with SHCN would result in reduced oral disease. Educational tools such as an informational DVD used in the present study are one of the ways in which the dental and medical fields can collaborate in the attempt to ensure persons with SHCN are receiving necessary oral health care. Virtual patient module with interactive multimedia which communicates and targets the need for dental student to decrease their perception of difficulty in caring for children with disabilities and to increase their competence is potentially an effective tool in meeting this need [28]. Also, in the present study, students indicated that the following would improve their ability to provide oral health care for persons with SHCN: further training, further education having equipment or products designed for persons with SHCN, continuing education related to oral health care for persons with SHCN, curriculum modification, and clinical case presentations. Similarly, a survey assessed the attitudes, behavior, and demographics of general dentists in US regarding their providing oral health care to patients with SHCN concluded that most general dentists surveyed in Nebraska see special needs patients of all ages and the most common reasons for not seeing more special needs patients were the level of the patient's disease, the patient's behavior, and insufficient training/experience [29].

Educating and instructing health care professionals, including dental students, are crucial before attentiveness and awareness can be extent to people. Programs to encourage and educate preventive procedures have been shown to increase awareness and knowledge as well as the ability to recall information related to health [30, 31]. Investigators concluded that video instruction and written materials are effective approaches and tools to increase health knowledge of parents and other adult learners [32]. In the present study, 20% of the students wish the presentation provided more information and videos and 15% of the students reported having informal training on oral health for person with SHCN which was in the form of some YouTube videos. A study tested an audio-visual aid for providing educational tool to caregivers concluded that audio-visual aid is powerful tool [33, 34]. Another study concluded that education of caregivers is important [35, 36]. A study reported effectiveness of oral health education of caregivers on special needs patients in form of a lecture, hands-on training, and a facilitated group discussion of those education methods [35, 36]. There is indication of national and international dissimilarity in the training and education at the undergraduate level which reflects in the content and quality of their education in SHCN [37]. This has been shown to correlate with the students' enthusiasm towards Dougall providing care for patients with SHCN in their future practice and their confidence SHCN [37]. Developing curriculum in Special Care Dentistry for dental students has been reported SHCN [37]. The importance and role of endorsing, encouraging, and harmonizing education in Special Care Dentistry as a means of reducing disparities in oral health was emphasized SHCN [20]. A study explored how Canadian and US dental schools instruct and educate students about individuals with SHCN and which challenges and goals for curricular modifications they recognize showed that dental schools had a varied approaches to educating and instructing dental students about SHCN patients and 91% of the programs covered this topic in their clinics [21]. However, only 64% have a separate course about SHCN and 37% had a special clinical area in their school for treating these patients [21]. A study evaluated the perceptions of medical and dental educators and their students on the sufficiency of clinical and didactic training to provide examination and service for person with SHCN reported that there is need for more clinical and didactic training and preparation of medical and dental school graduates in this area [38]. The responsibility in education of dental student in this area represent acknowledgment of educators for their role to ensure that new graduates have both the instruction and education as well as the desire to provide needed dental care to persons with SHCN [17]. The educational intervention used in this study may be a time and cost effective way to increase the knowledge of dental students on the unique aspects of oral health for persons with SHCN.

This study had some limitations; the most obvious were the sample size and the nature of the design and only male students in one dental college were included. However, this pilot study indicated some important information regarding knowledge and awareness of sophomore dental students regarding oral health care of persons with SHCN and the effectiveness of an education program on their knowledge. Another limitation that we have encountered is that all the data collected in this study was by self-reporting, therefore it was subject to recall bias which included intentional deception, poor memory, and misunderstanding questions. In addition, participants may not have answered questions due to incomplete information received from the educational intervention as some students reported that the presentation was fast which add to lack of clinical experience for sophomore dental students. Also, the presentation contains some dental terms which may be new to the sophomore students and some students have other concerns which reflected in their suggestions such as changes on the presentation and the need for more information and videos. In the same time it should be noted that this study only tested a short-term increase in knowledge among students and not long-term retention of the knowledge. Future study may assess more students and also at different levels as well as long-term retention of the knowledge by the students. Also, finding a way to assess participants' subsequent application of knowledge gained from the educational intervention would be very interesting.

## 5. Conclusion

Under the experimental conditions and within the limitations of this study, the following conclusions can be drawn.

1. Viewing the educational PowerPoint presentation with the video was effective in communicating and providing sophomore dental students with instructive basic information on persons with SHCN and resulted in significantly improved posttest scores.
2. Sixty percent of sophomore dental students self-rated their knowledge on persons with SHCN as being minimal before the educational intervention and only 10% and 15% reported having formal and informal training, respectively.
3. Ninety percent of the students reported that they were very likely or somewhat likely to use the information provided in the educational intervention.

## Figures and Tables

**Table 1:** Frequency of the answers in the pre- and posttest questionnaires.

Question number	Pretest			Posttest		
	Correct	Incorrect	Do not know	Correct	Incorrect	Do not know
1	15	4	1	19	0	1
2	15	5	0	16	4	0
3	14	4	2	18	2	0
4	5	1	14	19	1	0
5	4	8	8	16	4	0
6	9	1	10	12	6	2
7	11	3	6	16	3	1
8	2	14	4	3	16	1
9	7	6	7	15	3	2
10	8	6	6	9	8	3
11	2	4	14	10	5	5
12	4	7	9	11	6	3
13	8	3	9	16	1	3
14	2	7	11	11	7	2
15	5	1	14	10	6	4
16	14	2	4	17	1	2
17	17	0	3	17	1	2
18	15	0	5	16	1	3
19	15	1	4	17	1	2
20	7	4	9	12	4	4
21	6	8	6	14	4	2
22	3	11	6	15	3	2
23	8	3	9	11	5	4
24	10	2	8	13	5	4
25	4	5	11	9	8	3
26	7	3	10	12	4	4

Total	217	113	190	354	109	59

**Table 2:** The answers to the question “how would you rate your knowledge of oral health for person with SHCN” before and after the presentation/educational intervention.

Rate of knowledge	Before the presentation frequency (%)	After the presentation frequency (%)
Extensive	0 (0%)	1 (5%)
Moderate	1 (5%)	16 (80%)
Minimal	12 (60%)	3 (15%)
None	7 (35%)	0 (0%)

Total	20 (100%)	20 (100%)
